# Differentiation of Shock Thyroid on Enhanced Chest CT from Hashimoto’s Thyroiditis and Multinodular Goiter—A Pilot Study in Ten Patients with Shock Thyroid

**DOI:** 10.3390/jcm15166411

**Published:** 2026-08-19

**Authors:** Min Ji Son, Seung Min Yoo, Charles S White

**Affiliations:** 1 Department of Radiology, CHA University Bundang Medical Center, Bundang 13496, Republic of Korea; smj3006@naver.com; 2 Department of Radiology, University of Maryland, Baltimore, MD 21201, USA; cswhite999@gmail.com

**Keywords:** shock thyroid, CT, Hashimoto’s thyroiditis, multinodular goiter

## Abstract

**Background/Objectives:** The CT features of shock thyroid are enlarged thyroid glands with inhomogeneous enhancement and peri-thyroid fluid. However, if peri-thyroid fluid is absent or equivocal, shock thyroid may be confused with multinodular goiter or Hashimoto’s thyroiditis on enhanced chest CT. Thus, we investigated CT characteristics that differentiate shock thyroid from multinodular goiter or Hashimoto’s thyroiditis. **Methods**: We retrospectively identified 10 patients with shock thyroid who underwent enhanced chest CT and thyroid function tests (TFT) from March 1, 2025, to May 31, 2026. A control group consisted of 21 randomly selected patients with multinodular goiter (*n* = 10) or Hashimoto’s thyroiditis (*n* = 11). CT features, including capsular sparing with low attenuation, a prominent vessel sign, and a nodule with a sharp margin, were compared between groups. **Results:** There was a significant difference in the prevalence of capsular sparing with low attenuation between the shock thyroid and control groups [90.0% (9/10) versus 0.0% (0/21), *p* < 0.001]. There was also a significant difference in the frequency of a prominent vessel sign between the shock thyroid and Hashimoto’s thyroiditis groups [0.0% (0/10) versus 63.6% (7/11), *p* < 0.001], and a nodule with a sharp margin between the shock thyroid and multinodular goiter groups [0.0% (0/10) versus 80.0% (8/10), *p* < 0.001]. **Conclusions**: CT findings such as capsular sparing with low attenuation accompanied by peri-thyroid fluid collection may represent potential imaging markers of shock thyroid and require validation in a larger multicenter cohort.

## 1. Introduction

Shock thyroid is an extremely uncommon entity on CT scans. Only a few case reports have been published in the literature, and there has been no observational study [1,2,3,4,5,6,7]. Thus, the exact mechanism as well as prevalence and mortality regarding shock thyroid are unclear. Brochert et al. first reported shock thyroid in patients with hypovolemic shock. Based on previous case reports, the characteristic chest CT features of shock thyroid are enlarged thyroid glands with inhomogeneous enhancement and a peri-thyroid fluid collection, and a lack of previous history of direct neck trauma in patients with various types of shock. However, similar CT findings may be present in non-shock thyroid patients with Hashimoto’s thyroiditis or multinodular goiter. In prior case reports, a definitive diagnosis of shock thyroid relied on resolution of thyroid abnormalities on the follow-up CT [1,2,3,4,5]. In this context, the purpose of this study was to investigate typical CT findings of shock thyroid to differentiate it from multinodular goiter or Hashimoto’s thyroiditis.

## 2. Materials and Methods

### 2.1. Patients

**Figure 1:**
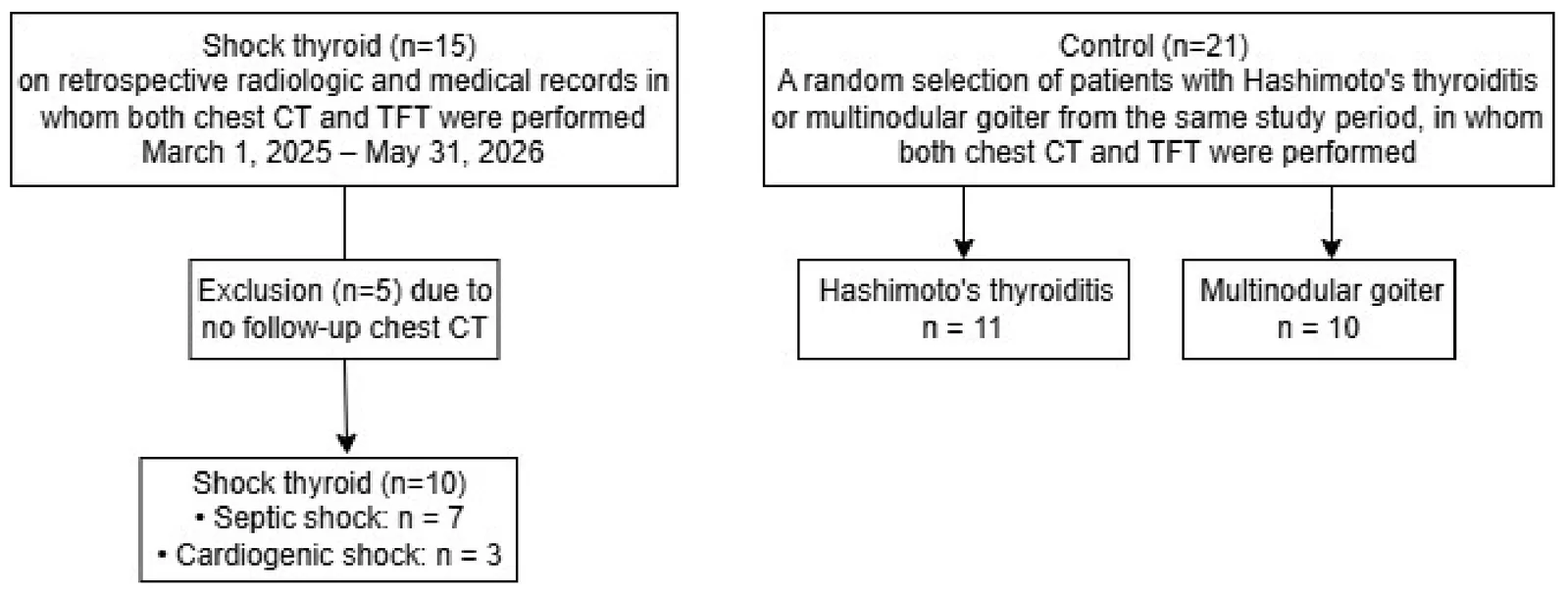
Flow chart of the patient selection process.

Using a retrospective search of radiologic reports and a chart review, we identified 15 patients with shock thyroid due to septic or cardiogenic shock in whom both enhanced chest CT and thyroid function tests (TFTs) were performed between 1 March 2025, and 31 May 2026. After excluding 5 patients who lacked follow-up chest CT, we ultimately included 10 patients (septic shock, *n* = 7 and cardiogenic shock, *n* = 3) with shock thyroid. Shock thyroid on enhanced chest CT was considered present when there were enlarged thyroid glands with inhomogeneous enhancement and/or peri-thyroid fluid collection without a history of neck trauma, and resolution of the thyroid abnormalities on follow-up CT [1]. For the control group, we randomly selected 21 patients during the same study period, consisting of 11 patients with Hashimoto’s thyroiditis and 10 patients with multinodular goiter, in whom enhanced chest CT and TFTs were performed within one month of each other. Median values of serum thyroid-stimulating hormone (TSH), triiodothyronine (T3), and free thyroxine (fT4) levels were compared between the shock thyroid group and the control group. This study was approved by the institutional review board and ethics committee (No: 2026-06-081). The detailed patient selection process and study design are summarized in the flow chart (Figure 1).

### 2.2. CT Protocols

Chest computed tomography (CT) examinations were performed using a 64-channel multidetector CT scanner (Optima CT660; GE Healthcare, Milwaukee, WI, USA). The *Z*-axis scan coverage extended from the lower neck to the upper abdomen. Contrast medium (Xenetix, 150 mL; Guerbet, Villepinte, France) was administered intravenously using an automated power injector. The acquisition and reconstruction parameters were as follows: a tube voltage of 100 or 120 kVp, a tube current of 180–200 mA, a collimation of 0.625 mm, and a reconstructed slice thickness of 2.5 mm.

### 2.3. CT Image Analysis

Two chest radiologists with 32 and 13 years experience in chest imaging analyzed the CT findings by setting a window width of 450 Hounsfield Unit (HU) and level of 30 HU. Any disagreements were resolved by consensus. We evaluated the following CT characteristics: the presence of capsular sparing with low attenuation, a prominent vessel sign within the thyroid gland, and a nodule with a sharp margin. Capsular sparing with low attenuation in the thyroid gland on enhanced chest CT was considered positive when normal high attenuation of thyroid glands is preserved at the subcapsular portion (i.e., within 2 mm from the outer margin of thyroid glands) on enhanced chest CT. This is contrasted to inhomogeneous low attenuation of inner portions of thyroid glands. A prominent vessel sign on CT was considered present when enlarged vessels (≥2 mm in diameter of vessel) with branching linear appearance were demonstrated within the thyroid glands, suggesting hypervascularity. A prominent vessel sign on CT was based on a previous study showing hypervascularity of the thyroid glands on ultrasonography in patients with Hashimoto’s thyroiditis [8]. We assumed that the low attenuation areas observed in typical shock thyroid and Hashimoto’s thyroiditis might exhibit ill-defined margins on CT. Therefore, the presence of at least one thyroid nodule with a sharp margin was considered to favor multinodular goiter over shock thyroid or Hashimoto’s thyroiditis. Additionally, the presence of a peri-thyroid fluid collection on CT was evaluated in all enrolled patients.

### 2.4. Statistical Analysis

Statistical analyses were performed using SPSS version 27.0. The Shapiro–Wilk test was employed to evaluate the normality of continuous variable distributions. Continuous variables (age, TSH, T3, and fT4) were expressed as medians with interquartile ranges, and categorical variables (sex, comorbidities, and all evaluated CT characteristics) were expressed as numbers and percentages. The Kruskal–Wallis test was used to compare continuous variables among the three groups, followed by Dunn’s test as a post hoc analysis for pairwise comparisons if a significant difference was observed. Categorical variables were compared using Fisher’s exact test due to the small sample size and low expected cell frequencies. For categorical variables showing significant differences, pairwise post hoc comparisons were conducted using Fisher’s exact test with Bonferroni correction. Interobserver agreement for the qualitative CT features between the two readers was assessed using Cohen’s kappa statistics. A *p*-value of less than 0.05 was considered statistically significant for the primary analyses, while a corrected threshold of 0.0167 (0.05/3) was applied for the categorical post hoc pairwise comparisons.

## 3. Results

The baseline demographic and clinical characteristics of the study population are summarized in Table 1. The median age of the patients demonstrated a statistically significant difference among the three groups (*p* < 0.001). Post hoc analysis revealed that patients in the Hashimoto’s thyroiditis group were significantly younger than those in both the shock thyroid and multinodular goiter groups. Although the proportion of female patients was higher in the Hashimoto’s thyroiditis and multinodular goiter groups compared to the shock thyroid group, this difference did not reach statistical significance (*p* = 0.258). Among the evaluated comorbidities, the prevalence of diabetes mellitus showed a statistically significant difference among the three groups (*p* = 0.031), with the shock thyroid group demonstrating a notably higher prevalence than the Hashimoto’s thyroiditis group. No significant differences were observed among the three groups regarding the prevalence of hypertension, chronic kidney disease, and malignancy (*p* > 0.05 for all). The specific etiologies of septic or cardiogenic shock in the shock thyroid group are delineated in Table 2. The median time interval between the initial and follow-up CT in patients with shock thyroid was 34.0 days (25.0–54.8 days, Table 1).

With respect to laboratory findings, initial thyroid function tests showed significant differences in serum TSH and T3 levels among the groups (*p* < 0.001 for both). Post hoc comparisons indicated that the Hashimoto’s thyroiditis group had significantly higher TSH levels than the multinodular goiter group. Furthermore, the shock thyroid group demonstrated significantly lower T3 levels compared to the multinodular goiter group. Serum fT4 levels did not differ significantly among the three groups (*p* = 0.132).

**Figure 2:**
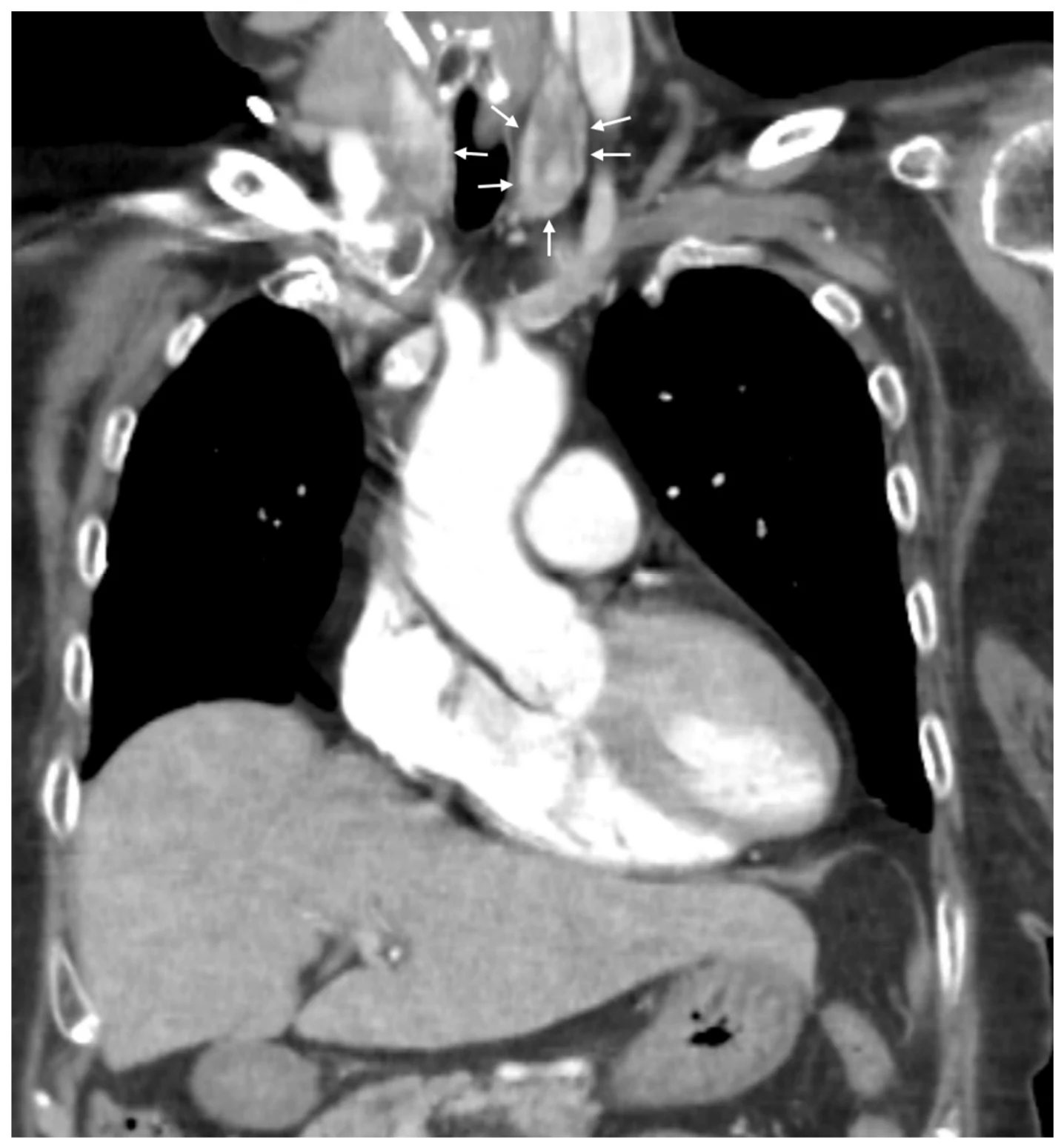
A case of probable shock thyroid in a 79-year-old woman. Note capsular linear sparing with low attenuation (arrows) in both thyroid glands. The enlarged thyroid glands show inhomogeneous enhancement. Thyroid function was normal in this patient on the same day as the CT.

**Figure 3:**
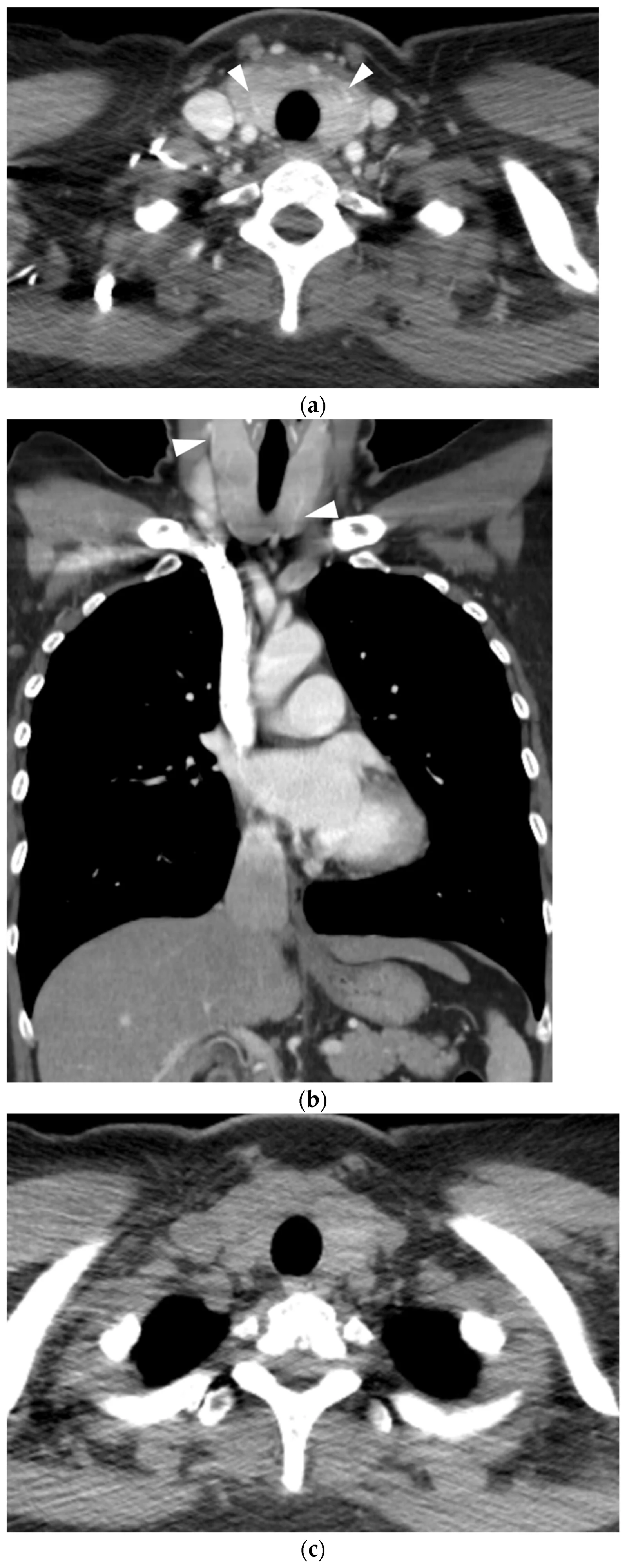
Representative case demonstrating Hashimoto’s thyroiditis on enhanced chest CT in a 50-year-old woman (**a**,**b**). Note diffuse thyroid enlargement with heterogeneous enhancement, but enhancing prominent vessels (arrowheads) are noted within the enlarged thyroid glands. This is a characteristic finding in patients with Hashimoto’s thyroiditis as compared to shock thyroid (**c**). Note diffuse low attenuation of enlarged thyroid glands on the pre-contrast CT image.

**Figure 4:**
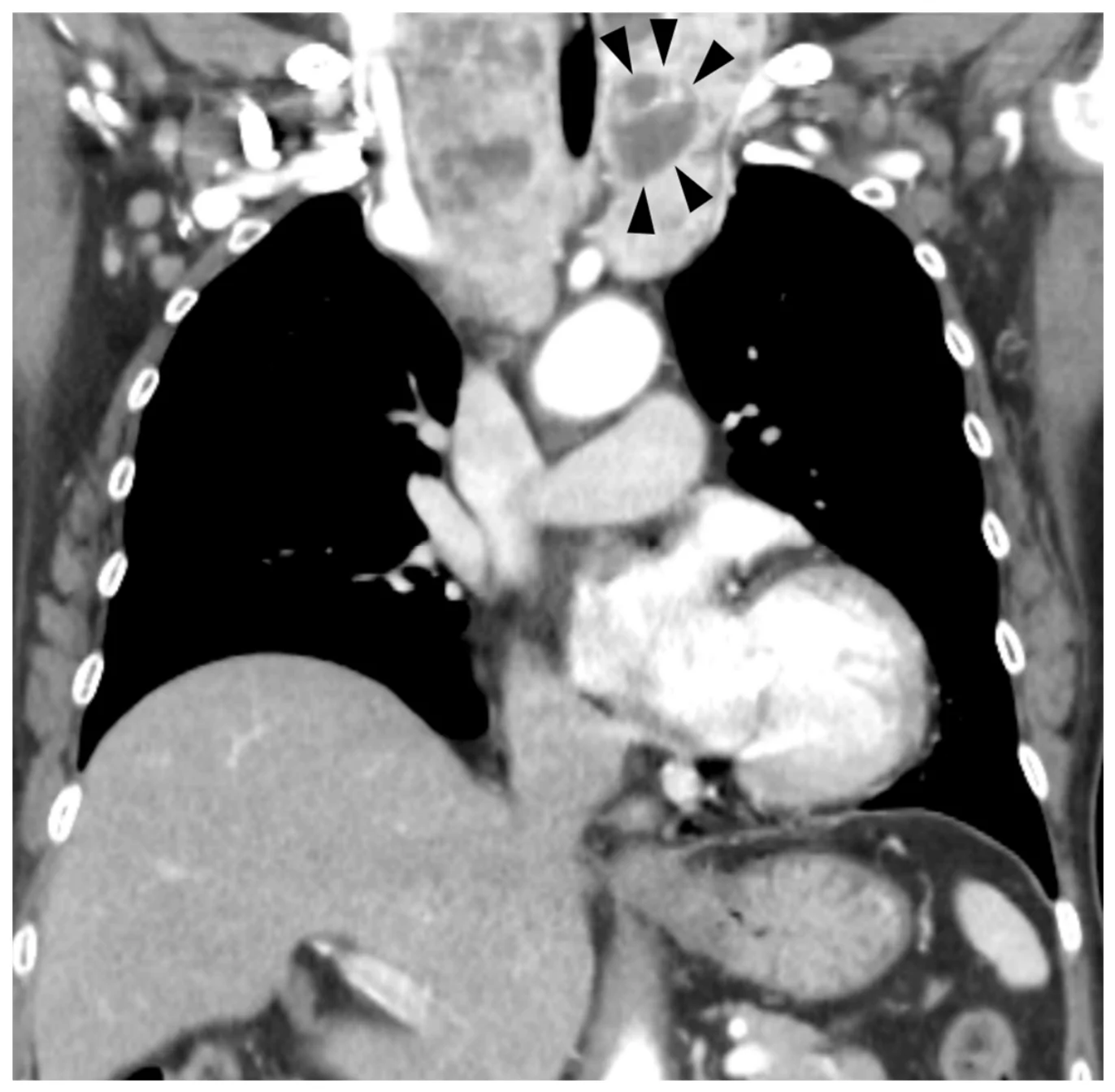
A case of multinodular goiter in a 66-year-old woman. Note multiple nodules in the enlarged thyroid glands. Some nodules in the left thyroid gland show sharp margination (arrowheads). This is a characteristic finding of multinodular goiter compared to shock thyroid on enhanced chest CT. TFTs were normal in this patient.

The enhanced chest CT features showed statistically significant differences among the three groups for all evaluated criteria (*p* < 0.001 for all by Fisher’s exact test, Table 3). Both capsular sparing with low attenuation (Figure 2) and a peri-thyroid fluid collection were exclusively observed in the shock thyroid group, whereas neither finding was present in the Hashimoto’s thyroiditis or multinodular goiter groups. Conversely, a prominent vessel sign (Figure 3) was exclusively identified within the Hashimoto’s thyroiditis group (*p* < 0.001). Additionally, the presence of a nodule with a sharp margin (Figure 4) was a characteristic feature unique to the multinodular goiter group. Interobserver agreement was excellent for all evaluated CT features between the two readers (kappa = 1.00).

## 4. Discussion

Previous studies have indicated that the typical features of shock thyroid on enhanced CT include the presence of enlarged thyroid glands with inhomogeneous enhancement and a peri-thyroid fluid collection [1,2,3,4,5,6,7]. However, doubt persists as to whether shock thyroid can be reliably differentiated from preexisting comorbidities (e.g., multinodular goiter or Hashimoto’s thyroiditis) without a follow-up CT. Indeed, in most previous case reports, the diagnosis of shock thyroid was supported by findings on follow-up CT, demonstrating a return to normal size and attenuation of the thyroid glands [1,2,3]. However, it may be beneficial for radiologists to identify characteristic CT findings of shock thyroid without the need for a follow-up examination. Notably, as the clinical presentation of shock may be equivocal, prompt identification of shock thyroid on CT may be helpful for the ED physician to identify compensated shock at the time of CT scanning (i.e., impending or concealed shock). In addition, discrimination of shock thyroid from other pathologies such as Hashimoto’s thyroiditis or multinodular goiter may obviate unnecessary downstream testing.

The previous case reports only indicated that CT findings of shock thyroid were enlarged thyroid glands with inhomogeneous attenuation and/or peri-thyroid fluid collection [1,2,3,4,5]. According to previous definition of shock thyroid, Hashimoto’s thyroiditis or multinodular goiter should be excluded by using follow-up CT. In contrast, the authors provide more specific CT features of shock thyroid, leading to more confident diagnosis of shock thyroid without follow-up CT scan. Specifically, the main findings in this study were that capsular sparing with low attenuation in the thyroid glands and a peri-thyroid fluid collection are typical CT findings of shock not demonstrated in Hashimoto’s thyroiditis or multinodular goiter. We hypothesize that capsular sparing with low attenuation in the thyroid glands may result from relative resistance of the thyroid capsule in the setting of the reperfusion injury due to its more solid structure compared to the inner thyroid parenchyma. Alternatively, it may be caused by increased vulnerability to ischemia in the deeper portion of the thyroid gland as compared to subcapsular portion, analogous to myocardial ischemia [9]. Peri-thyroid fluid collection was present exclusively in the shock thyroid group in this study (80%, 8/10). Peri-thyroid fluid collection may also be present in patients with direct traumatic injury on the neck. However, there was no patient with a history of traumatic injury at the presentation in the ED in our study. In contrast, a prominent vessel sign and nodule with a sharp margin on CT were only identified on Hashimoto’s thyroiditis and multinodular goiter, respectively. Visualization of a prominent vessel sign on CT in patients with Hashimoto’s thyroiditis may be caused by thyroid CT hypervascularity. This CT feature corresponds to the ultrasound finding of thyroid inferno [10]. A similar CT finding may also be identified in patients with Graves’ disease. However, we believe that Graves’ disease can be differentiated from shock thyroid based on clinical and laboratory findings [11,12].

There were f patients with Hashimoto’s thyroiditis who lacked a prominent vessel sign in this study. The size of the thyroid glands was normal or only slightly increased in these patients, suggesting a chronic stage or mild form of Hashimoto’s thyroiditis. Furthermore, most patients with multinodular goiter (80.0%, 8/10) demonstrated at least one thyroid nodule with a sharp margin on CT. This may serve as a useful CT finding to differentiate multinodular goiter from shock thyroid without the need for a follow-up CT.

Based on our study results, clinical and laboratory findings may also support differentiation of shock thyroid from Hashimoto’s thyroiditis or multinodular goiter. The shock thyroid group demonstrated significantly lower T3 levels compared to the multinodular goiter group in our study. This laboratory finding is consistent with a non-thyroidal illness syndrome (NTIS) pattern, wherein critical illness or acute shock states induce an initial time-dependent fall in circulating T3 levels as an adaptive physiological response to conserve systemic energy catabolism [13]. In addition, as Hashimoto’s thyroiditis is common in young or middle-aged women, the median age was younger in patients with Hashimoto’s thyroiditis compared to those with shock thyroid in this study. There were more patients with diabetes mellitus in the shock thyroid group compared to those with Hashimoto’s thyroiditis in this study. This may be caused by age differences between the two groups (i.e., vulnerability complicating septic or cardiogenic shock in older patients with diabetes mellitus versus resistance in younger patients with Hashimoto’s thyroiditis without diabetes mellitus).

This study has several limitations. First, there are a small number of patients which may lead to potential selection bias. Notably, only patients with septic or cardiogenic shock who underwent both chest CT and TFT screening were included in this study. In addition, this is a single-center retrospective study. The proposed imaging findings should therefore be considered preliminary, and await further validation. Second, the time interval between the initial and follow-up CT scan was variable in this study. Third, there were multiple potential confounders among the study population such as differences in age and comorbidities between groups, and the effect of clinical severity and the time elapsed since the onset of shock on the CT findings. However, the small sample size precluded logistic regression analysis in this study. Further studies with larger groups of enrolled patients and a prospective design are required to address this issue. Fourth, qualitative analysis was only performed in this study. This is a definite weak point of this study. However, the small size of capsular sparing, and the ill-defined margin and small size of inhomogeneous low attenuation in the inner portion of thyroid glands on shock thyroid made quantitative measurement of CT HU nonreproducible in this study. Fifth, in our study, Hashimoto’s thyroiditis and multinodular goiter were selected as the control group. However, several other thyroid conditions such as subacute thyroiditis, acute suppurative thyroiditis, diffuse thyroid edema, and infiltrative thyroid disease were not represented in this study, which is a limitation.

## 5. Conclusions

CT findings such as capsular sparing with low attenuation accompanied by peri-thyroid fluid collection may represent potential imaging markers of shock thyroid and require validation in larger multicenter cohort.

## Figures and Tables

**Table 1:** Baseline demographic, clinical characteristics, and comorbidities of the study groups.

Characteristics	Shock Thyroid (*n* = 10)	Hashimoto’s Thyroiditis (*n* = 11)	Multinodular Goiter (*n* = 10)	*p*-Value *
Age (years)	77.5 (68.2–78.8) ^a^	50.0 (44.0–53.5)	67.0 (63.5–72.0) ^a^	<0.001
Sex (Female)	6 (60.0%)	10 (90.9%)	8 (80.0%)	0.258
Comorbidities				
hypertension	5 (50.0%)	3 (27.3%)	6 (60.0%)	0.322
diabetes mellitus	6 (60.0%) ^a^	1 (9.1%)	2 (20.0%)	0.031
chronic kidney disease	2 (20.0%)	1 (9.1%)	1 (10.0%)	1
Malignancy	3 (30.0%)	3 (27.3%)	6 (60.0%)	0.264
Thyroid Function Test				
TSH (normal: 0.27–4.2µIU/mL)	1.76 (0.70–3.34)	4.81 (2.51–10.11) ^b^	0.03 (0.01–0.26)	<0.001
T3 (normal: 0.8–2.0 ng/mL)	0.66 (0.55–0.79] ^b^	1.05 (0.84–1.17)	1.22 (1.12–1.39)	<0.001
Free T4 (normal: 0.92–1.68 ng/dL)	1.15 (1.02–1.48)	1.01 (0.85–1.22)	1.49 (1.20–1.54)	0.132
Time interval for CT f/u (days)	34.0 (25.0–54.8)	N/A	N/A	

Note: Data are presented as medians (interquartile ranges, 25th–75th percentiles) for continuous variables and as numbers (percentages) for categorical variables. * *p*-values were calculated using the Kruskal–Wallis test for continuous variables and Fisher’s exact test for categorical variables. ^a^ Statistically significant difference (*p* < 0.05) versus the Hashimoto’s Thyroiditis group in post hoc pairwise comparisons. ^b^ Statistically significant difference (*p* < 0.05) versus the Multinodular Goiter group in post hoc pairwise comparisons. Abbreviations: TSH, thyroid-stimulating hormone; T3, triiodothyronine; T4, thyroxine.

**Table 2:** Specific etiologies of septic or cardiogenic shock in the shock thyroid group (*n* = 10).

Category/Specific Cause	Number of Cases (%)
Septic shock	7 (70%)
Pneumonia	2 (20%)
Infectious colitis/Colitis	2 (20%)
Acute pyelonephritis (APN)	1 (10%)
Pancytopenia with secondary sepsis	1 (10%)
Unknown origin	1 (10%)
Cardiogenic shock/Cardiac arrest	3 (30%)
Asphyxia-induced arrest	1 (10%)
Unknown origin	2 (20%)
Total	10 (100%)

**Table 3:** Comparison of enhanced chest CT features among the study groups.

CT Characteristics	Shock Thyroid (*n* = 10)	Hashimoto’s Thyroiditis (*n* = 11)	Multinodular Goiter (*n* = 10)	*p*-Value
Capsular sparing with low attenuation	9 (90.0%)	0 (0.0%)	0 (0.0%)	<0.001
Peri-thyroid fluid collection	8 (80.0%)	0 (0.0%)	0 (0.0%)	<0.001
Prominent vessel sign	0 (0.0%)	7 (63.6%)	0 (0.0%)	<0.001
Nodule with a sharp margin	0 (0.0%)	0 (0.0%)	8 (80.0%)	<0.001

## Data Availability

The data supporting the findings of this study are available from the corresponding author upon reasonable request, due to privacy or ethical restrictions.

## Abbreviations

The following abbreviations are used in this manuscript:

TFT	Thyroid Function Test
TSH	Thyroid-Stimulating Hormone
T3	Triiodothyronine
fT4	Free Thyroxine
